# Supplementary material for: Metabolic modeling of the International Space Station microbiome reveals key microbial interactions
Source: Microbiome. 2022 Jul 6;10:102. doi: 10.1186/s40168-022-01279-y (PMC9258157; doi:10.1186/s40168-022-01279-y)
Supplement: Supplementary file 2 — Additional file 1: Supplementary Methods M1. Genome-scale metabolic reconstruction and modeling. [file 40168_2022_1279_MOESM1_ESM.pdf]

Supplementary Methods M1

Genome-scale metabolic network reconstruction

The genome sequences were, either via direct import from the NCBI RefSeq database or via upload in the case of GenBank genomes, as input to KBase [1] to reconstruct automated genome-scale metabolic models (GSMMs). These GSMMs capture the metabolic network of these microorganisms to the extent known across published literature and reaction databases [2]. The bacterial genomes were first annotated with the RASTtk toolkit v1.073 [3–5]. The metabolic models were then reconstructed using the Build Metabolic Model v2.0.0 app, and a Gram-positive/Gram-negative template was appropriately selected for reconstruction. The gap-filling was done with a minimal medium readily available in the KBase public media database, RefGlucoseMinimal, and increasing the maximum uptake of Glucose to 10 mmol/gDW-h. For the reconstruction of fungal models, the Build Fungal Model v1.0.0 was used, templates were selected from the available fourteen templates, and gap-filling was also carried out with the default settings.

The KBase [1] database demands that genomes are annotated in a specific format. Some genomes were incompatible with KBase [1], owing to a lack of annotation or improper format. Therefore, in such cases, the reference genomes of the microorganisms were used instead of the whole-genome sequence from the ISS.

These models were used for both graph-theoretic analyses and constraint-based analyses. Constraint-based model artifacts, such as the reaction node for biomass, the nodes corresponding to the compounds for biomass, DNA replication, RNA transcription, and Protein biosynthesis, were removed from all the graphs. Supplementary Table S2 contains information on the reconstructions. All the models used are available from the GitHub repository: https://github.com/RamanLab/Metabolic-Modelling-of-the-ISS-Microbiome

Choice of methodology

We chose SteadyCom over other methods since dFBA [6] methods such as COMETS [7] or DFBAlab [8] require additional parameter details such as specific growth rate, V_{max}, and K_m for the particular substrates used for growth, without which the simulations cannot be carried out. Since this information is not available for our dataset, we chose to use steady-state FBA methods. Further, microbial abundances can be calculated using SteadyCom [9], which other methods like cFBA [10] do not provide. SteadyCom [9] is also scalable to larger microbial communities, and the runtime for the algorithm is relatively less; moreover, it has been very widely used to study microbial communities.

References

1. Arkin AP, Cottingham RW, Henry CS, Harris NL, Stevens RL, Maslov S, et al. KBase: The United States department of energy systems biology knowledgebase. Nature Biotechnology. 2018;36:566–9.

2. Thiele I, Palsson B. A protocol for generating a high-quality genome-scale metabolic reconstruction. Nature Protocols. 2010;5:93–121.

3. Brettin T, Davis JJ, Disz T, Edwards RA, Gerdes S, Olsen GJ, et al. RASTtk: A modular and extensible implementation of the RAST algorithm for building custom annotation pipelines and annotating batches of genomes. Scientific Reports. 2015;5:1–6.

4. Overbeek R, Olson R, Pusch GD, Olsen GJ, Davis JJ, Disz T, et al. The SEED and the Rapid Annotation of microbial genomes using Subsystems Technology (RAST). Nucleic Acids Research. 2014;42:D206–14.

5. Aziz RK, Bartels D, Best A, DeJongh M, Disz T, Edwards RA, et al. The RAST Server: Rapid annotations using subsystems technology. BMC Genomics. 2008;9:75.

6. Henson MA, Hanly TJ. Dynamic flux balance analysis for synthetic microbial communities. IET Systems Biology. 2014;8:214–29.

7. Dukovski I, Bajić D, Chacón JM, Quintin M, Vila JCC, Sulheim S, et al. A metabolic modeling platform for the computation of microbial ecosystems in time and space (COMETS). Nature Protocols 2021 16:11. 2021;16:5030–82.

8. Gomez JA, Höffner K, Barton PI. DFBAlab: A fast and reliable MATLAB code for dynamic flux balance analysis. BMC Bioinformatics. 2014;15:1–10.

9. Chan SHJ, Simons MN, Maranas CD. SteadyCom: Predicting microbial abundances while ensuring community stability. PLoS Computational Biology. 2017;13:e1005539.

10. Khandelwal RA, Olivier BG, Röling WFM, Teusink B, Bruggeman FJ. Community flux balance analysis for microbial consortia at balanced growth. PloS one. 2013;8.
