# Supplementary material for: Metabolic modeling of the International Space Station microbiome reveals key microbial interactions
Source: Microbiome. 2022 Jul 6;10:102. doi: 10.1186/s40168-022-01279-y (PMC9258157; doi:10.1186/s40168-022-01279-y)
Supplement: Supplementary file 3 — Additional file 2: Supplementary Figure S1. BSL-2 pathogens. The word cloud shows the dominant and persistent BSL-2 pathogens at each location in each flight. [file 40168_2022_1279_MOESM2_ESM.pdf]

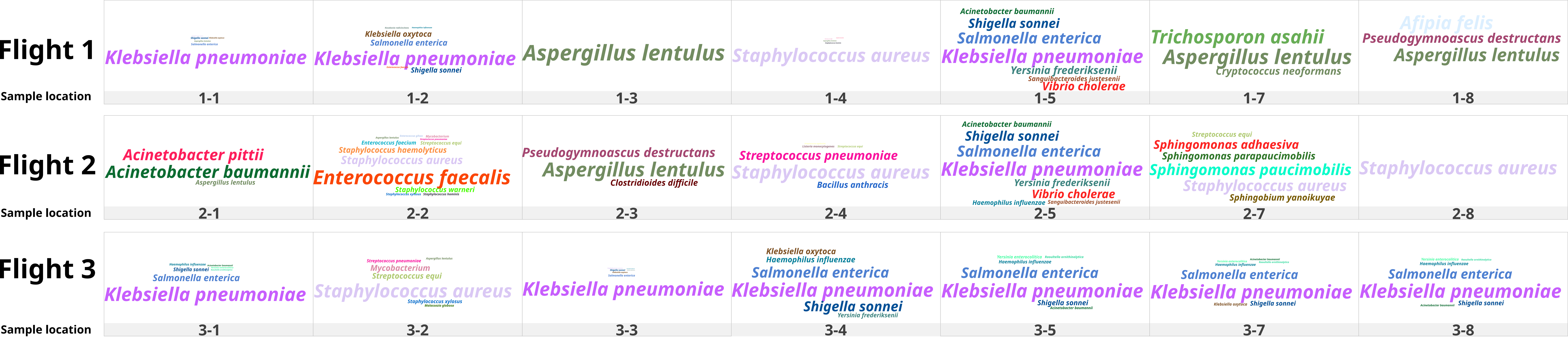

This word cloud was generated using the ISS data mentioned in Singh et al. 2018 [1]. A table consisting of human pathogenic microbes was created, and the table was uploaded to Megan6 software [2]. The "taxon chart window" was used to visualize the abundance distribution of the taxa as a word cloud. All the species having >1% of the total reads in consideration were visualized in the word cloud.

1. Singh NK, Wood JM, Karouia F, Venkateswaran K: Succession and persistence of microbial communities and antimicrobial resistance genes associated with International Space Station environmental surfaces. *Microbiome* 2018, **6**:204

2. Huson DH, Beier S, Flade I, Gorska A, El-Hadidi M, Mitra S, Ruscheweyh HJ, Tappu R: MEGAN Community Edition - Interactive Exploration and Analysis of Large-Scale Microbiome Sequencing Data. *PLoS Comput Biol* 2016, **12**:e1004957
