# Supplementary figures and images for: Metabolic modeling of the International Space Station microbiome reveals key microbial interactions
Source: Microbiome. 2022 Jul 6;10:102. doi: 10.1186/s40168-022-01279-y (PMC9258157; doi:10.1186/s40168-022-01279-y)

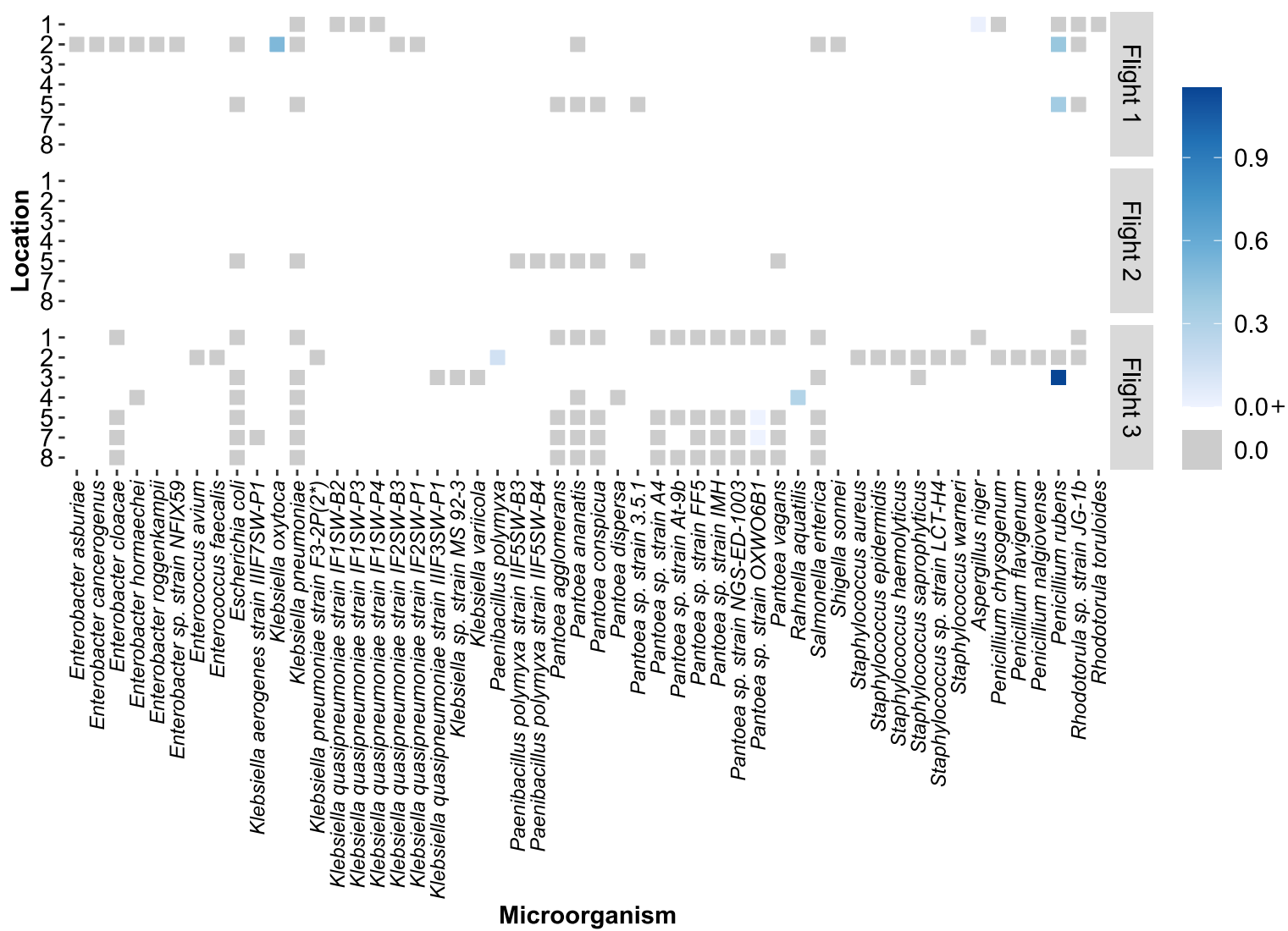

Supplement: Supplementary file 4 — Additional file 3: Supplementary Figure S2. Extent of metabolic benefit conferred by an individual microorganism to its coexisting microorganisms. The heatmap depicts the range of Community Support Indices (${CSI}_{A\to \tilde{A}}$CSIA→A~) that indicate the metabolic support rendered by an individual microorganism to its coexisting microorganisms, by virtue of it being in that location. On the X-axis is the list of microorganisms in consideration, and on the Y-axis is the flight number and the concerned location number. A darker blue tile indicates the microorganism is highly beneficial. [file 40168_2022_1279_MOESM3_ESM.pdf]

## L1

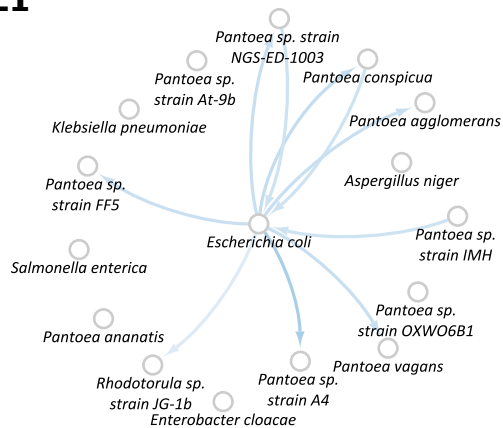

## L5

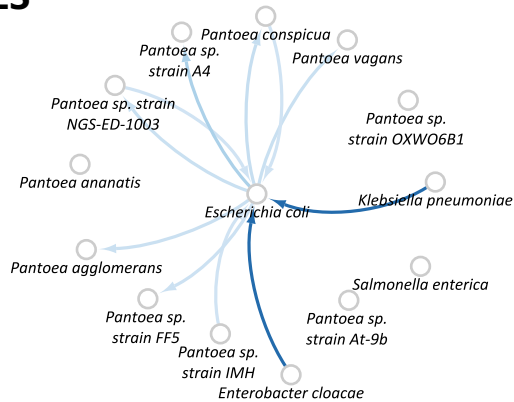

### L3

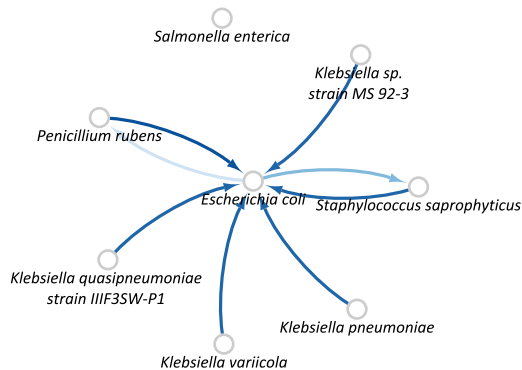

## L7

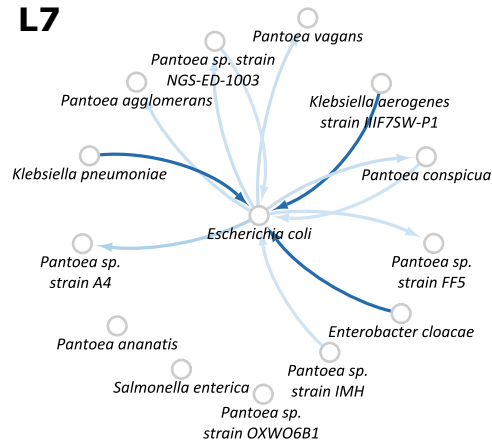

## L4

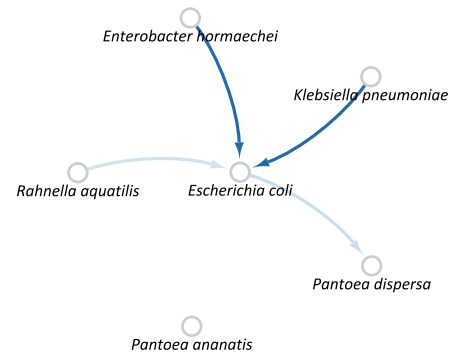

## L8

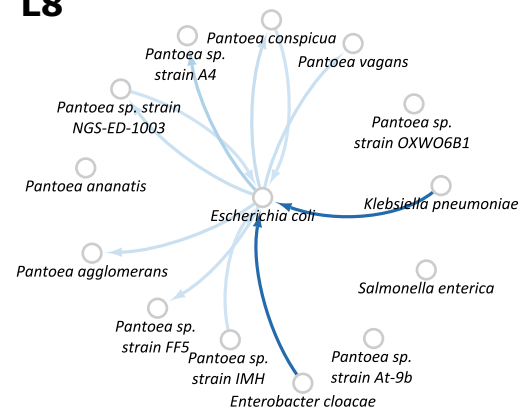

## Legend

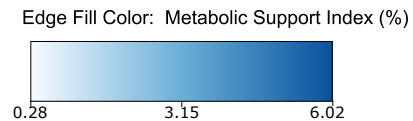

Supplement: Supplementary file 5 — Additional file 4: Supplementary Figure S3. Microbial association networks depicting the metabolic dependencies of E. coli and its coexisting microorganisms on each other, during Flight 3. Cytoscape was used to construct and visualize these networks across all locations during Flight 3 (F3). The nodes are labelled with microorganisms that co-inhabit that location with E. coli. The directed edges are directed from the metabolically supportive microorganism to the metabolically dependent microorganism. The color of these directed edges are mapped to the Metabolic Support Indices (MSI), represented as percentages, such that the gradient from light blue to dark blue represents an increasing MSI. [file 40168_2022_1279_MOESM4_ESM.pdf]

L1

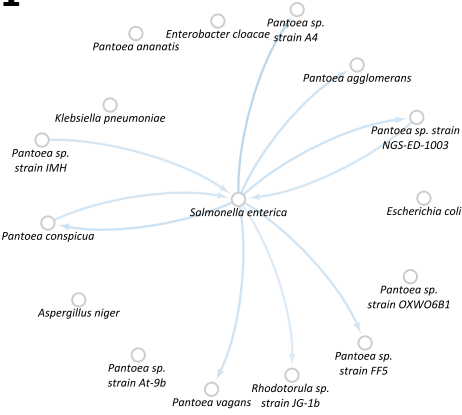

L3

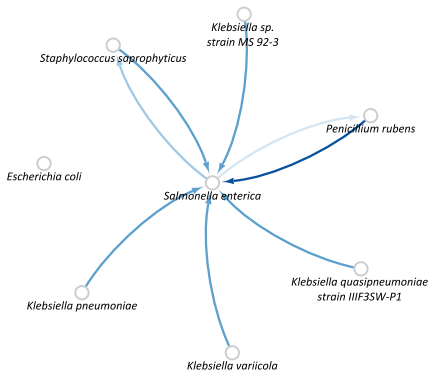

L5

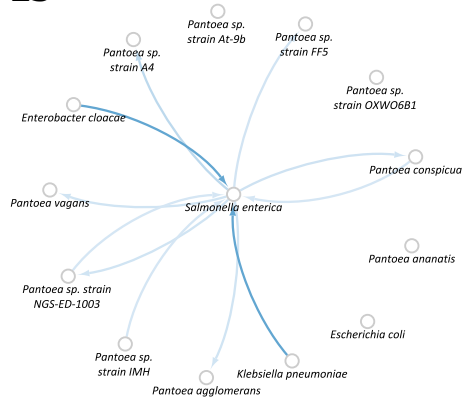

L7

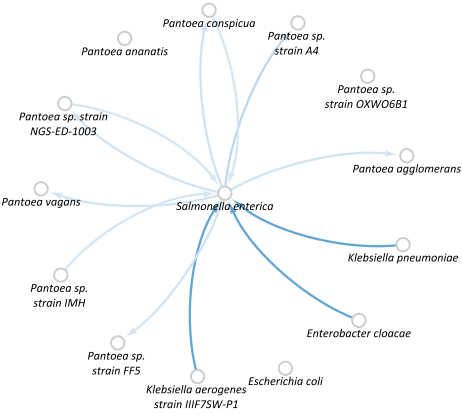

L8

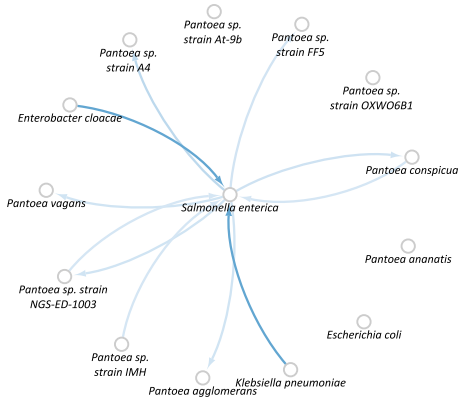

Legend

Edge Fill Color: Metabolic Support Index (%)

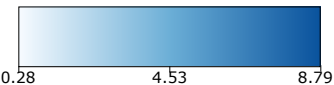

Supplement: Supplementary file 6 — Additional file 5: Supplementary Figure S4. Microbial association networks depicting the metabolic dependencies of S. enterica and its coexisting microorganisms on each other, during Flight 3. Cytoscape was used to construct and visualize these networks across all locations during Flight 3 (F3). The nodes are labelled with microorganisms that co-inhabit that location with S. enterica. The directed edges are directed from the metabolically supportive microorganism to the metabolically dependent microorganism. The color of these directed edges is mapped to the Metabolic Support Indices (MSI), represented as percentages, such that the gradient from light blue to dark blue represents an increasing MSI. [file 40168_2022_1279_MOESM5_ESM.pdf]

Figure S5A

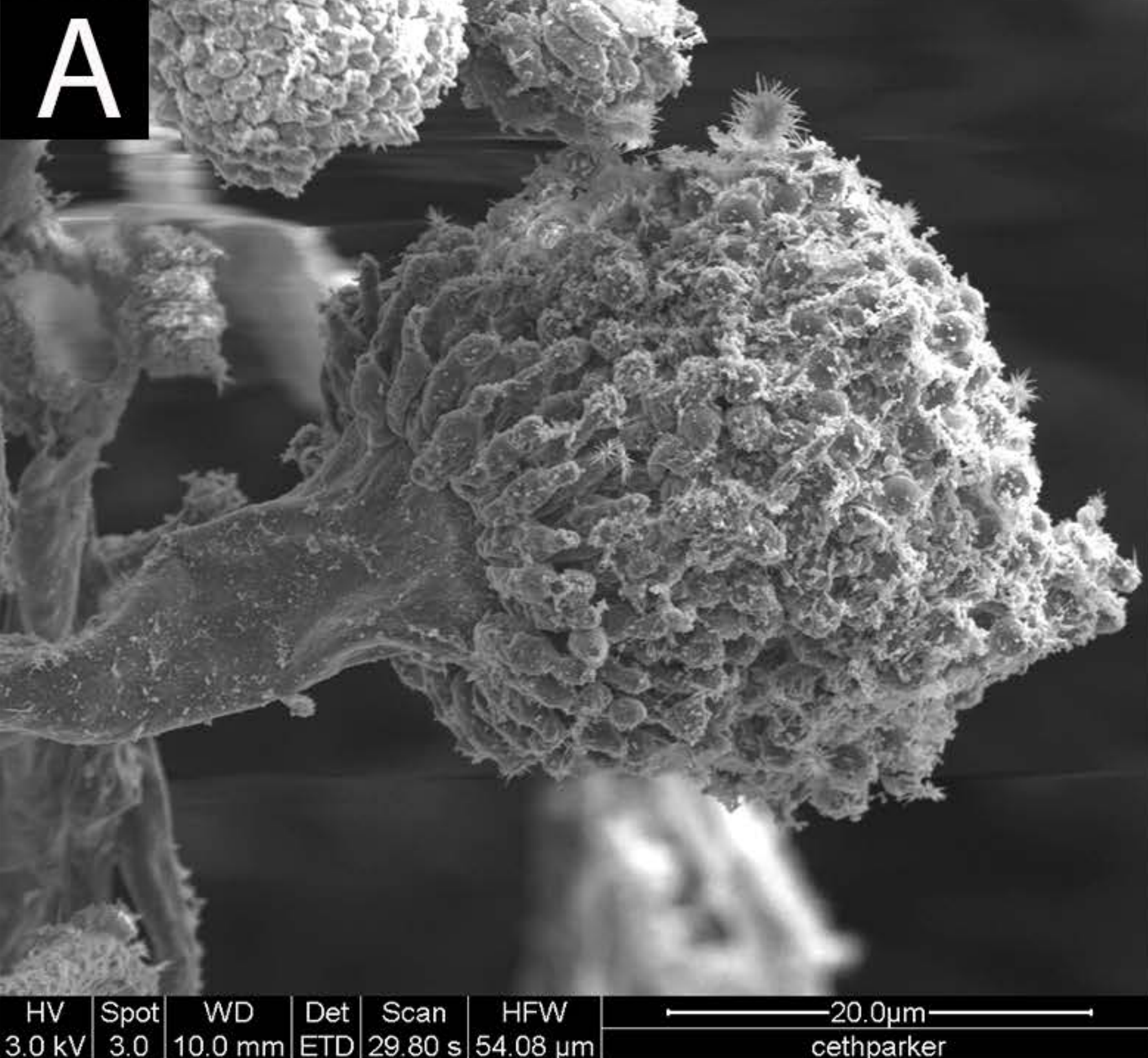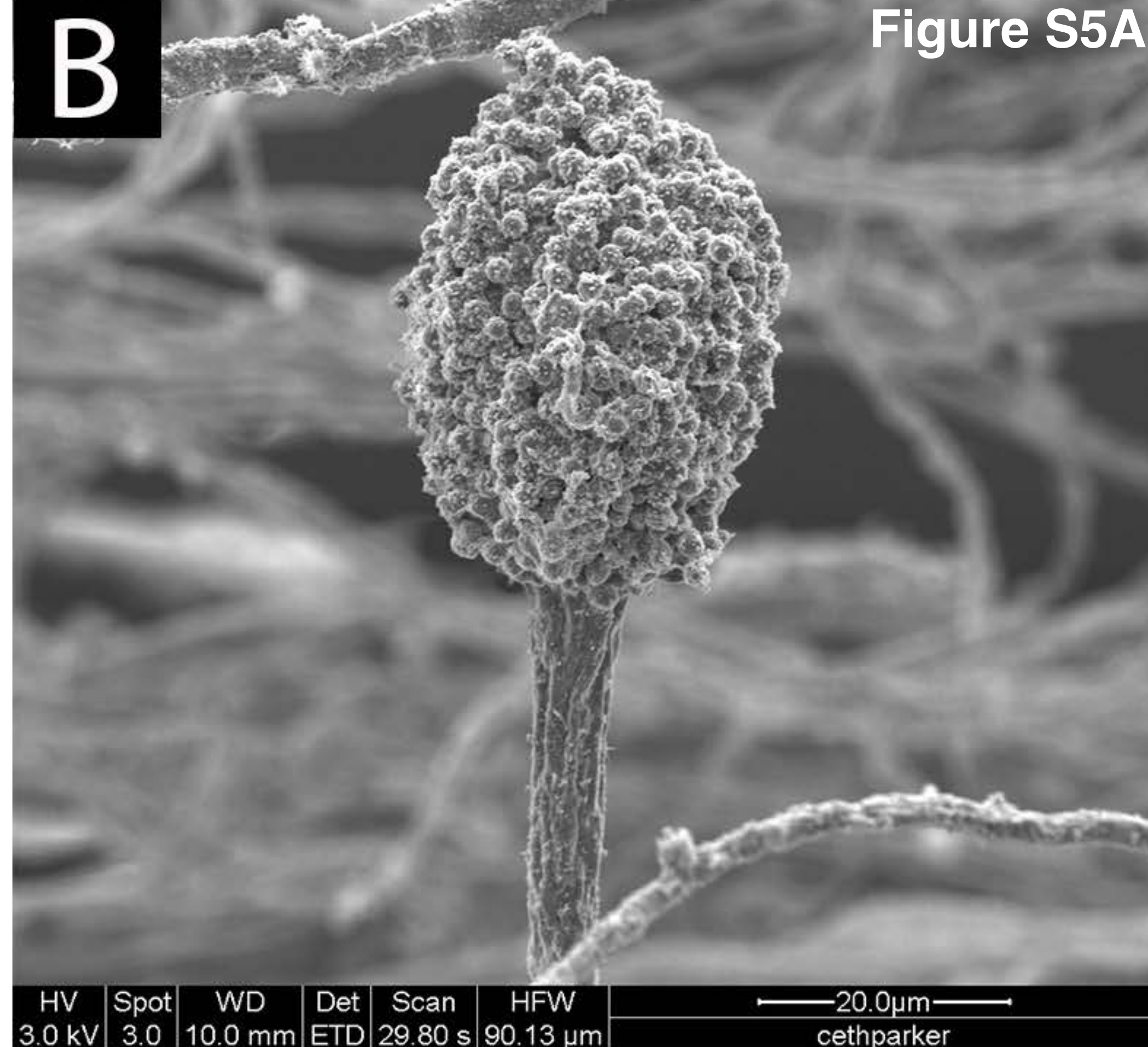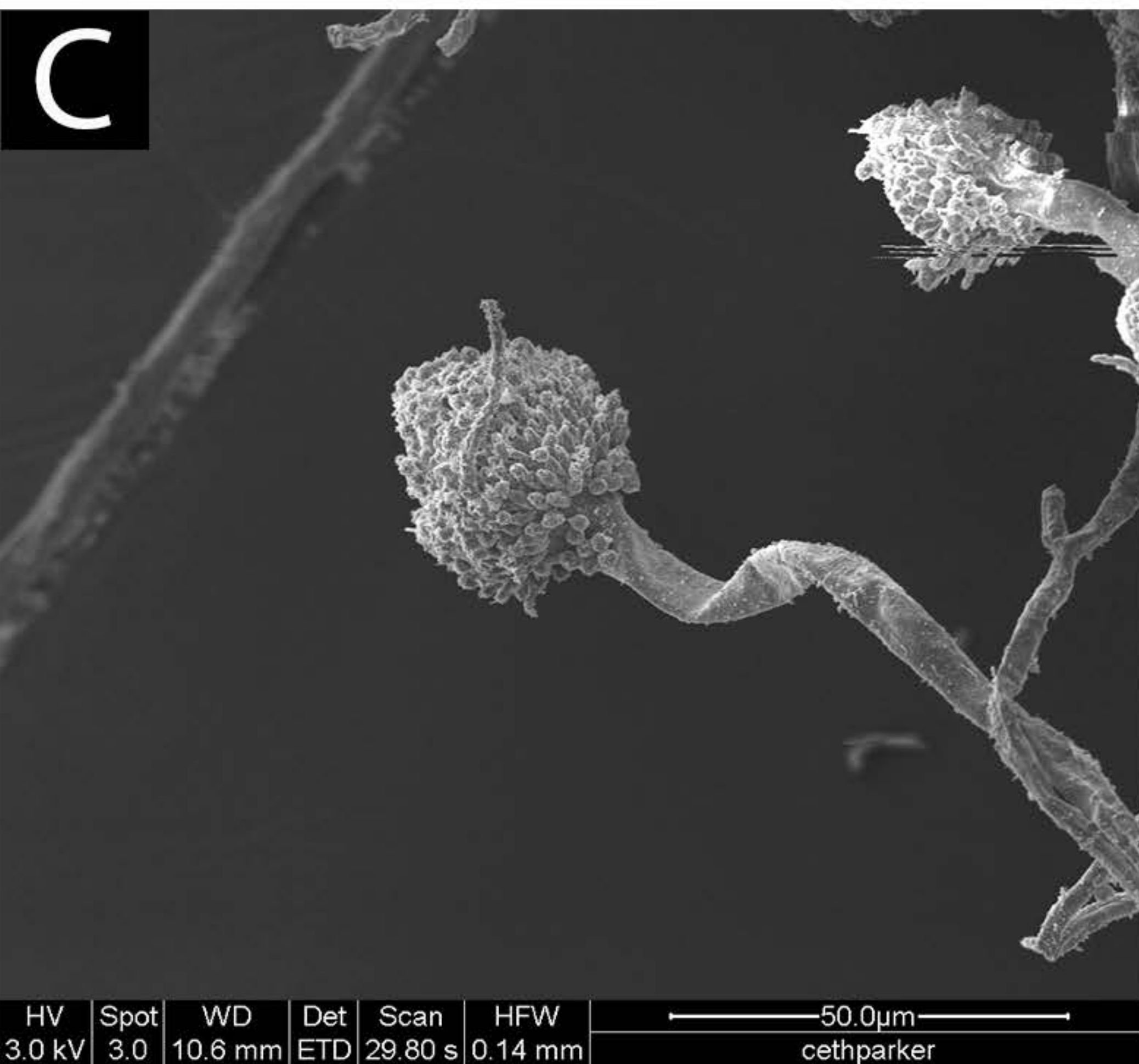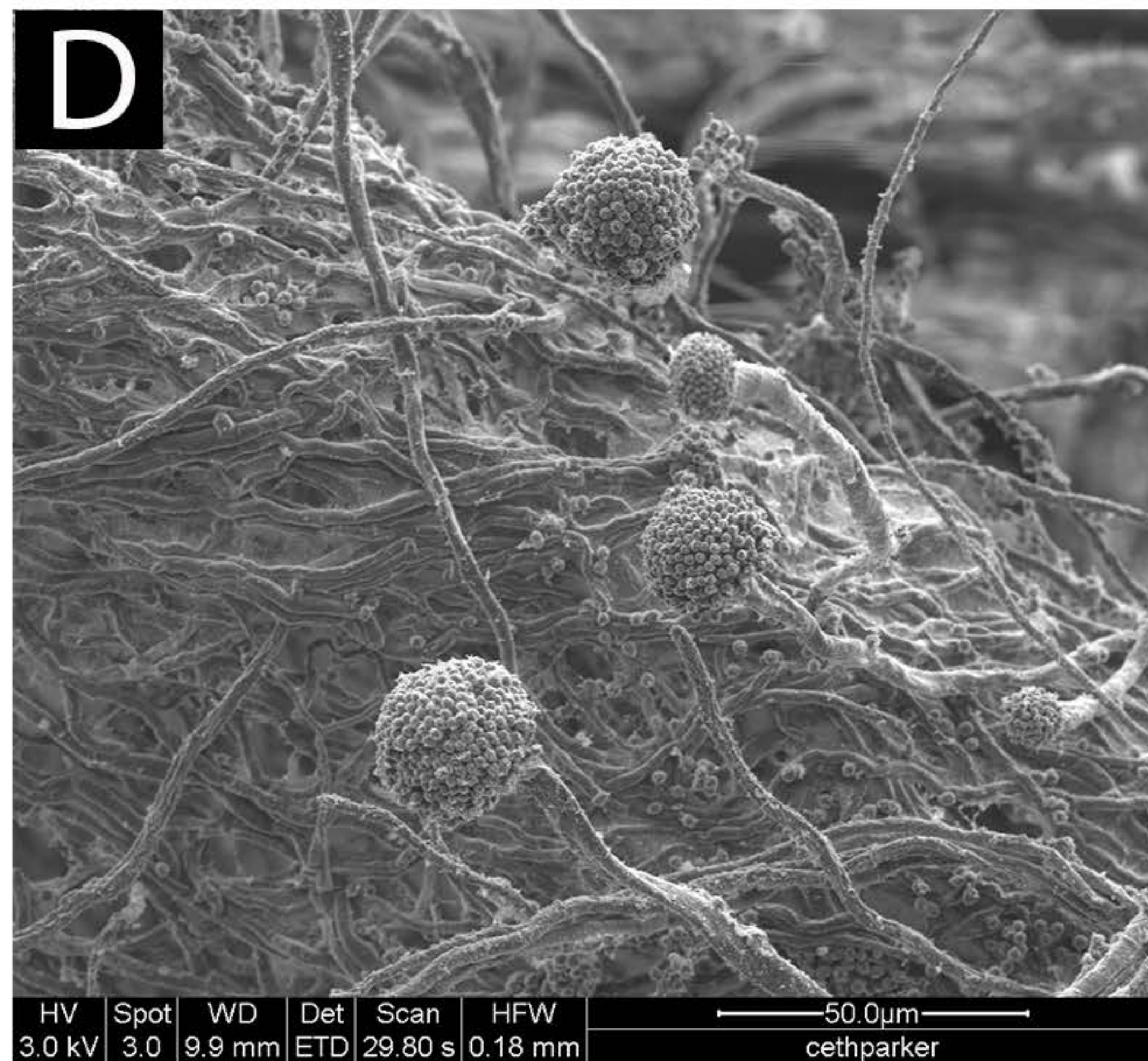

Figure S5B

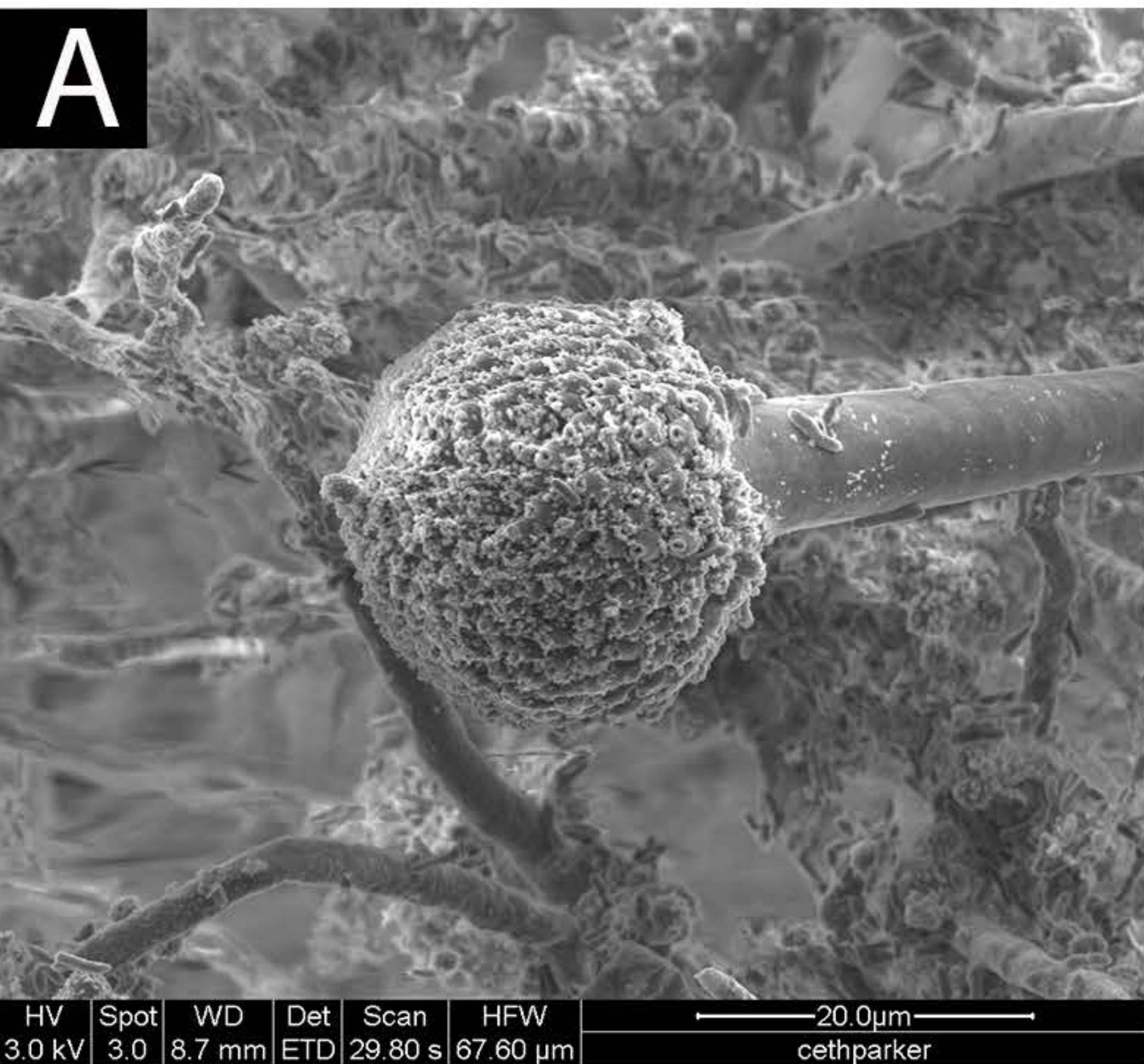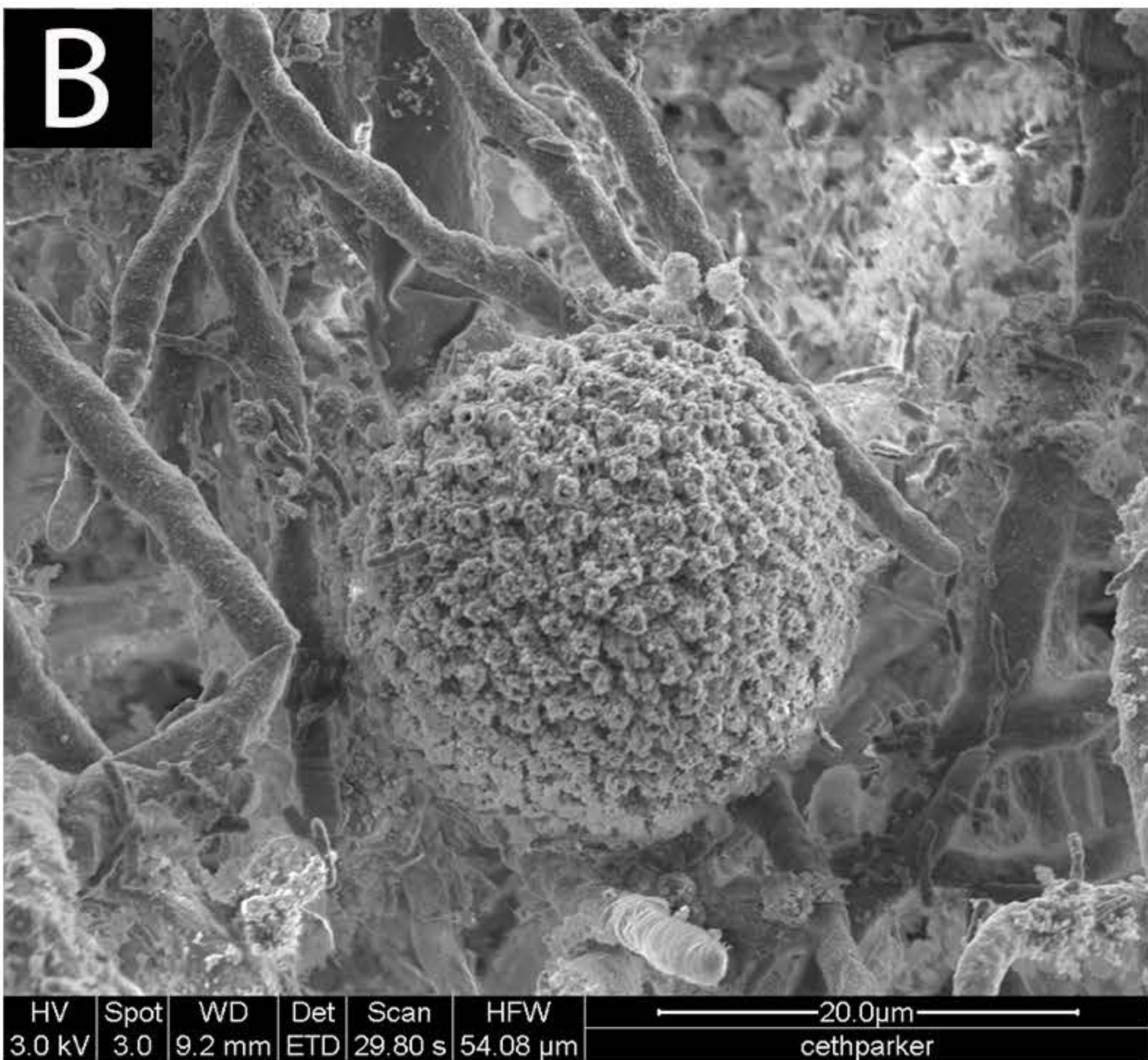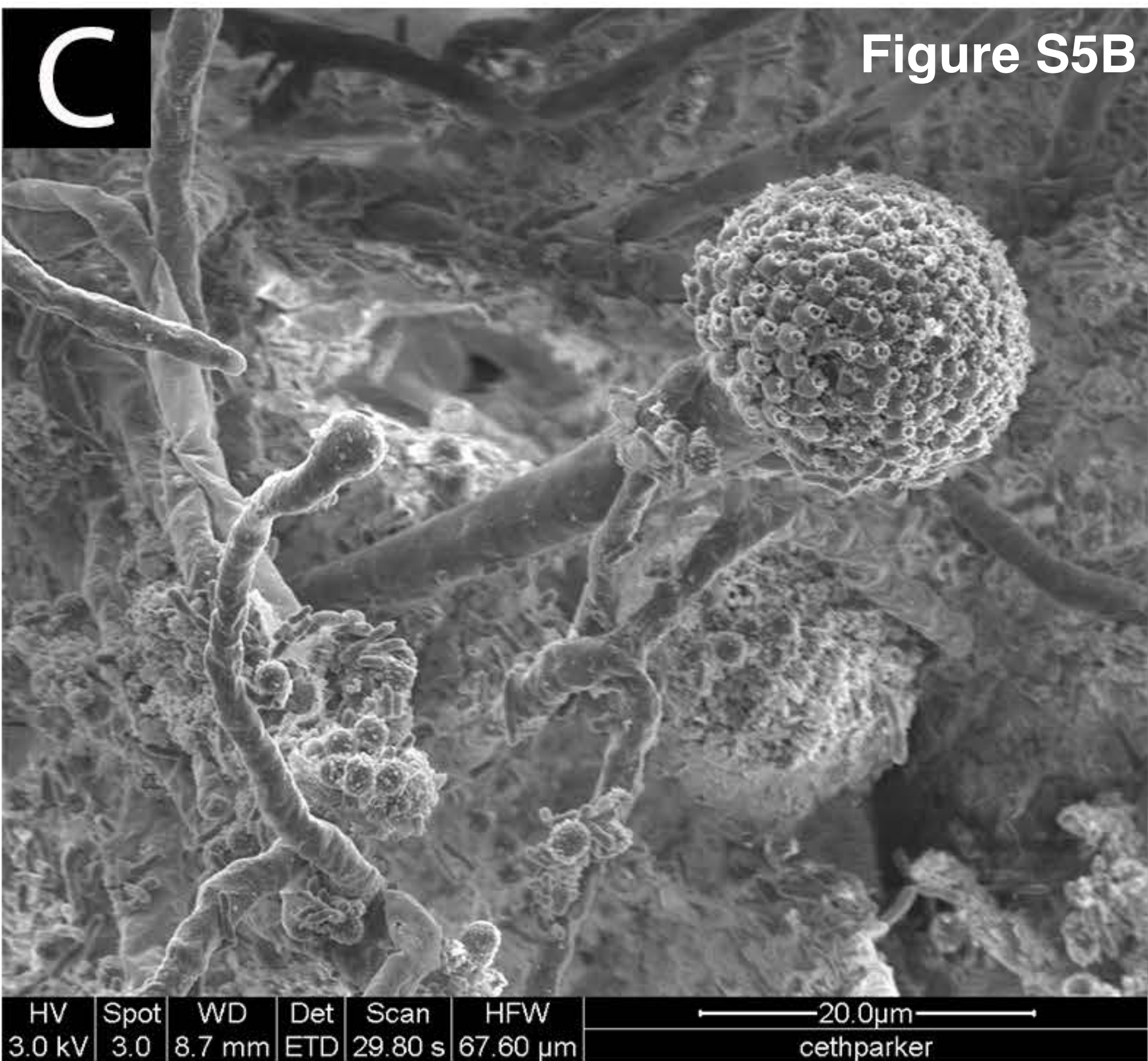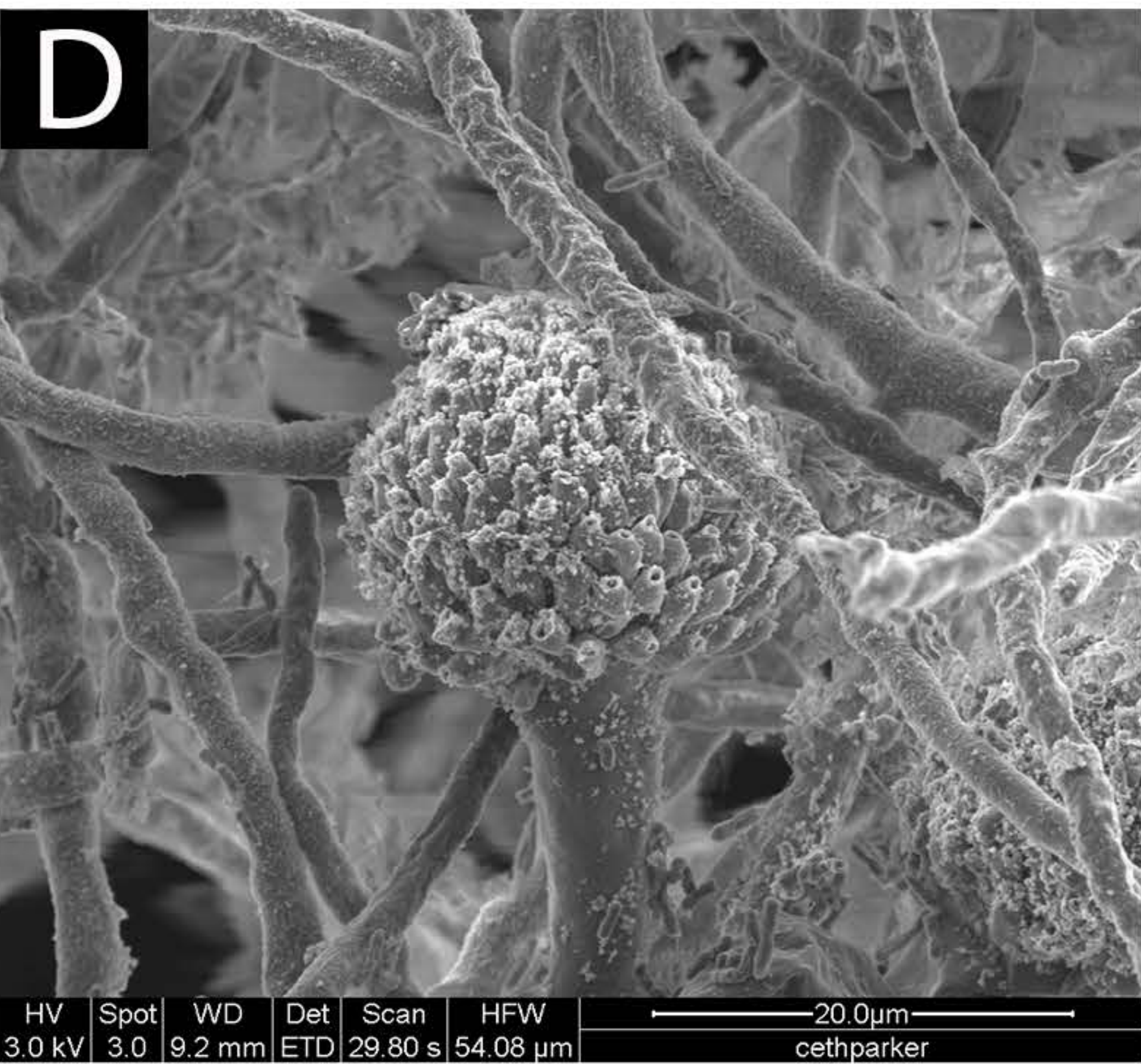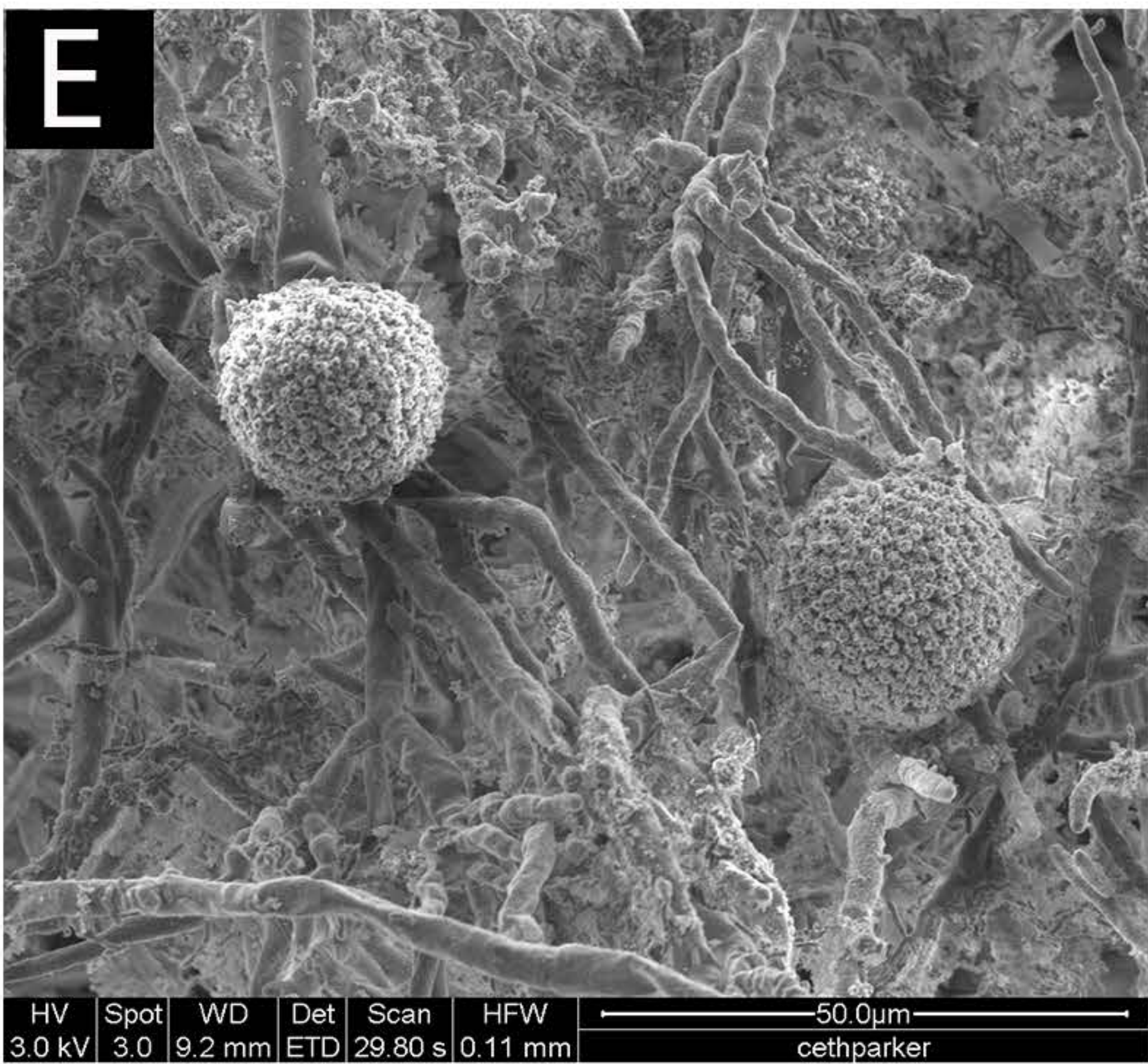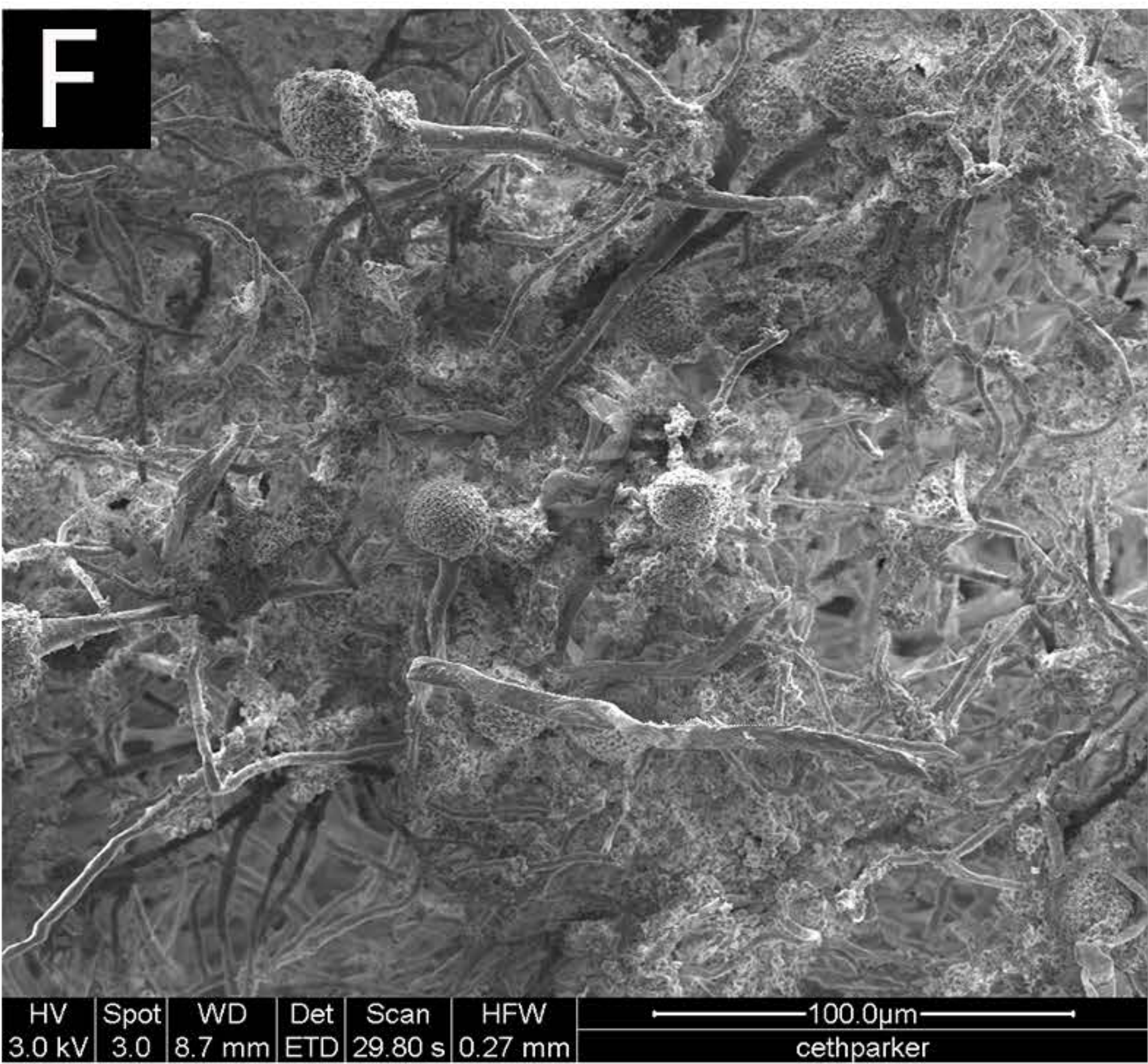

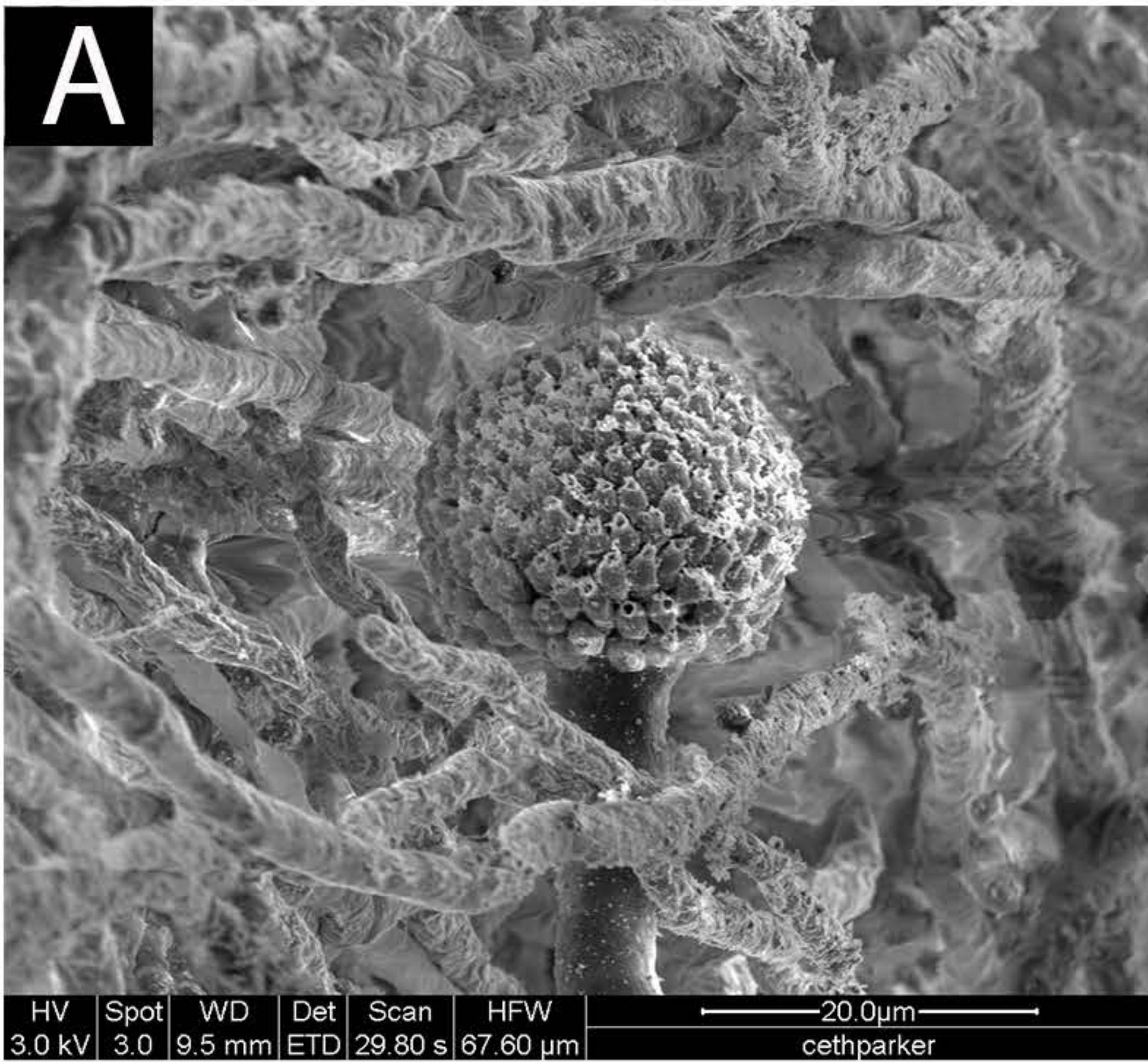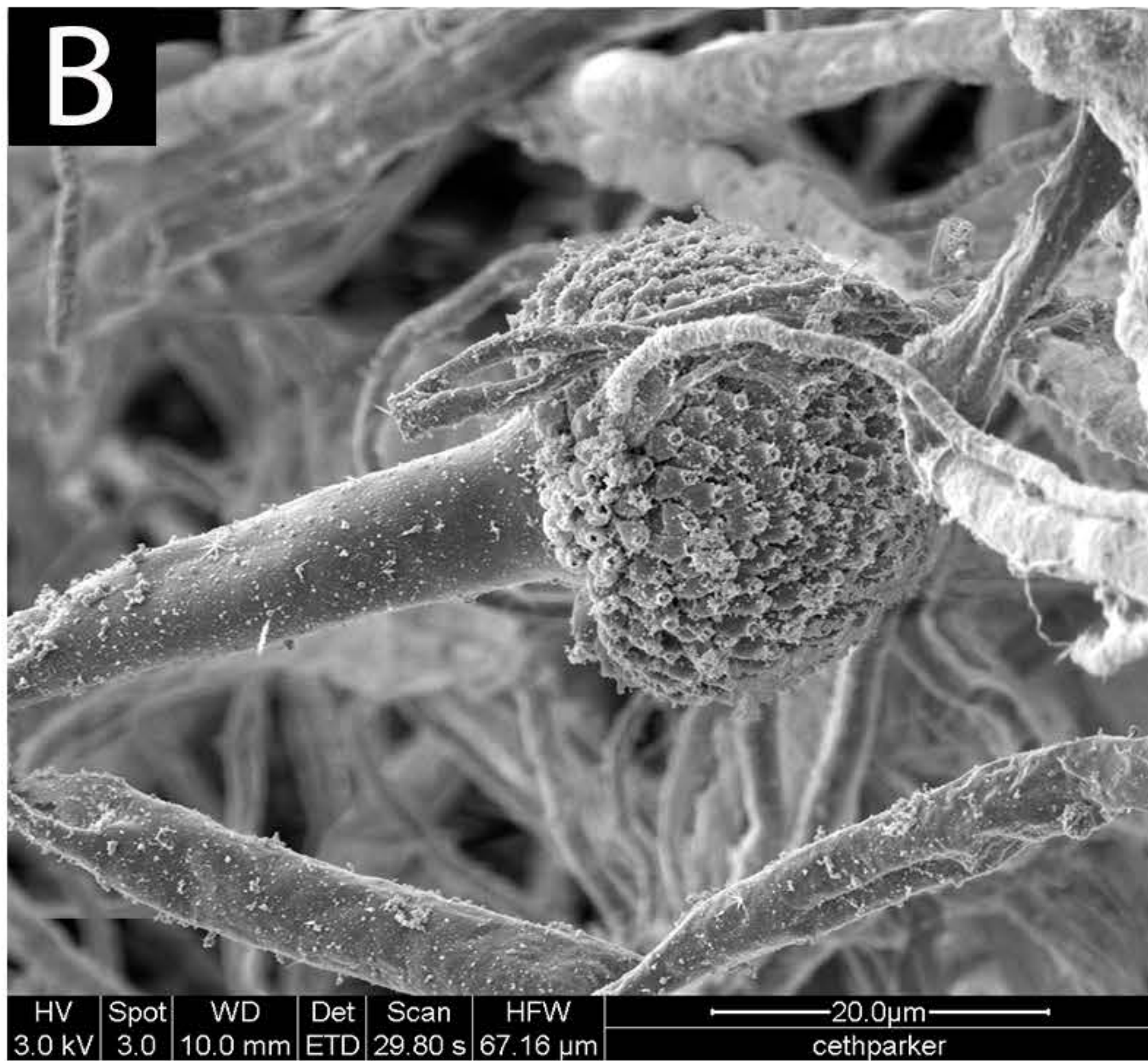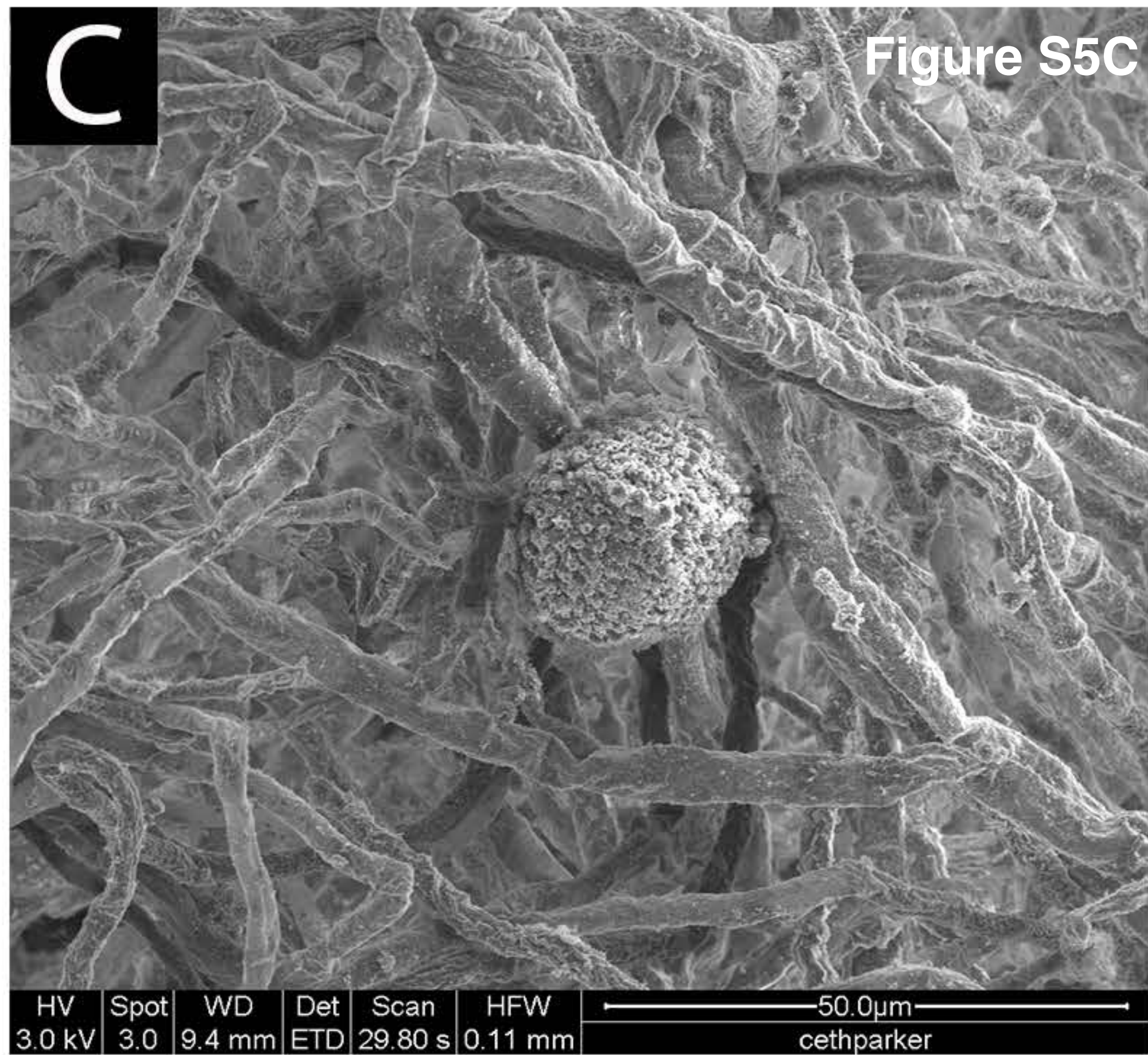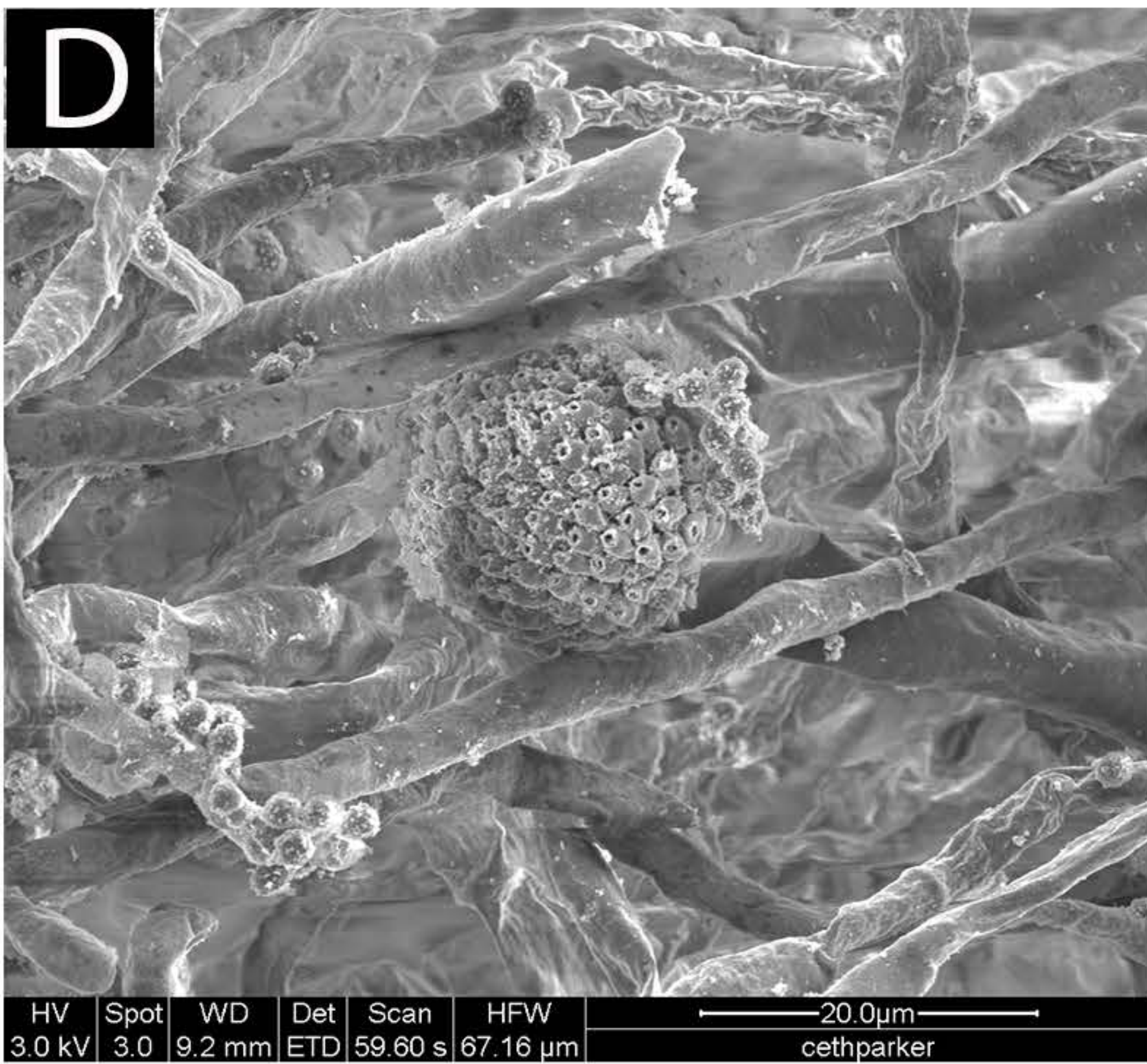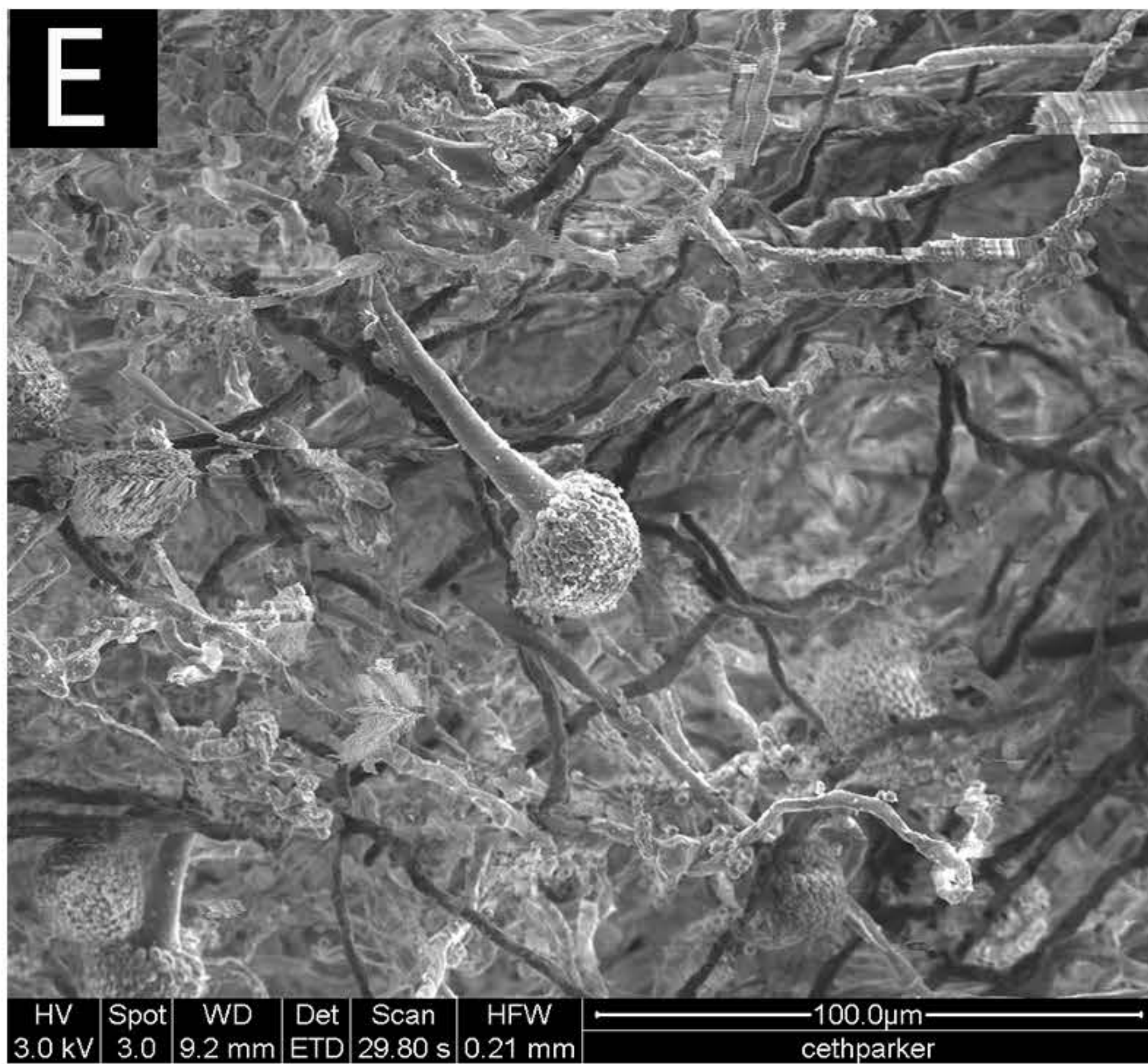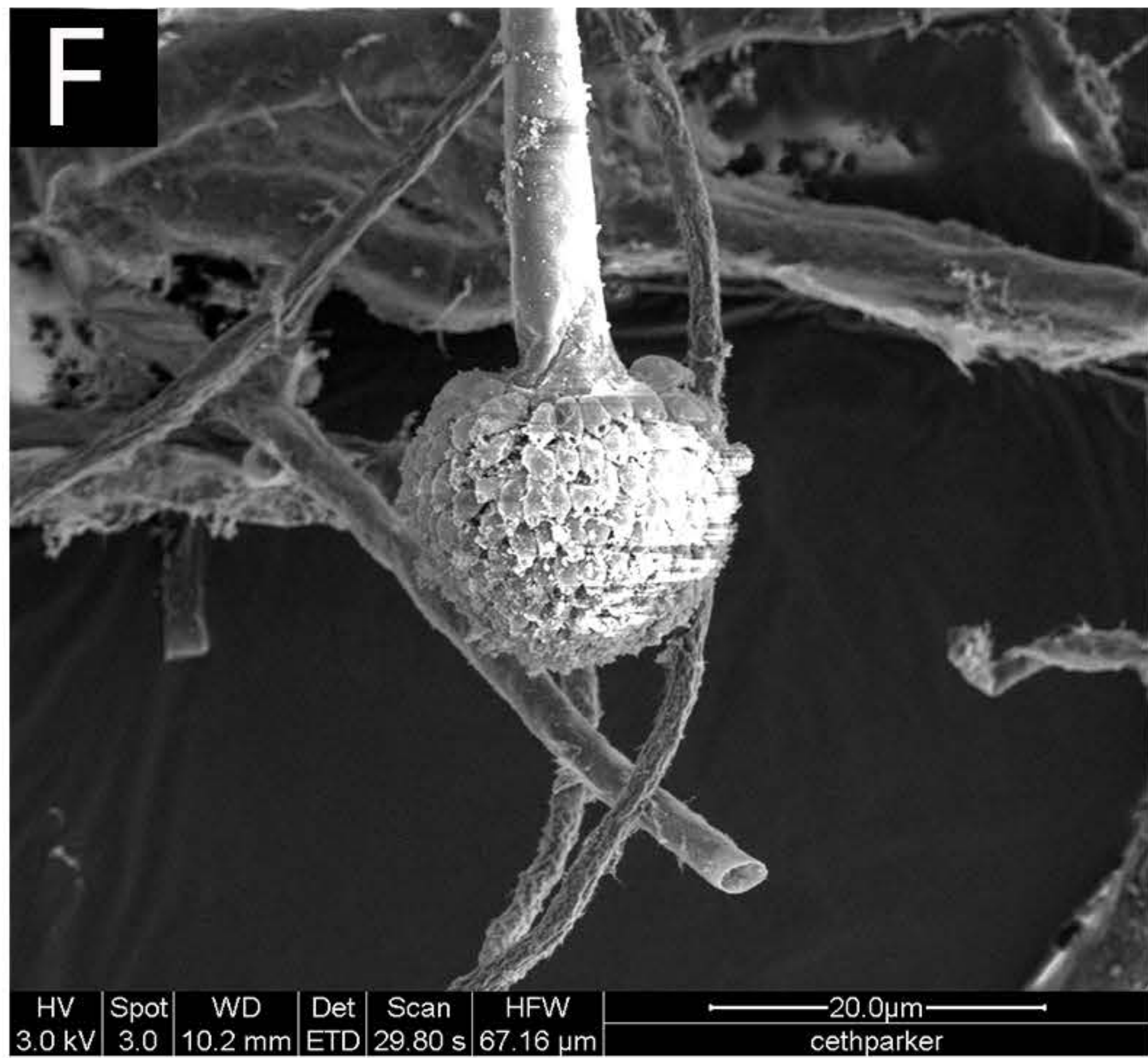

Figure S5D

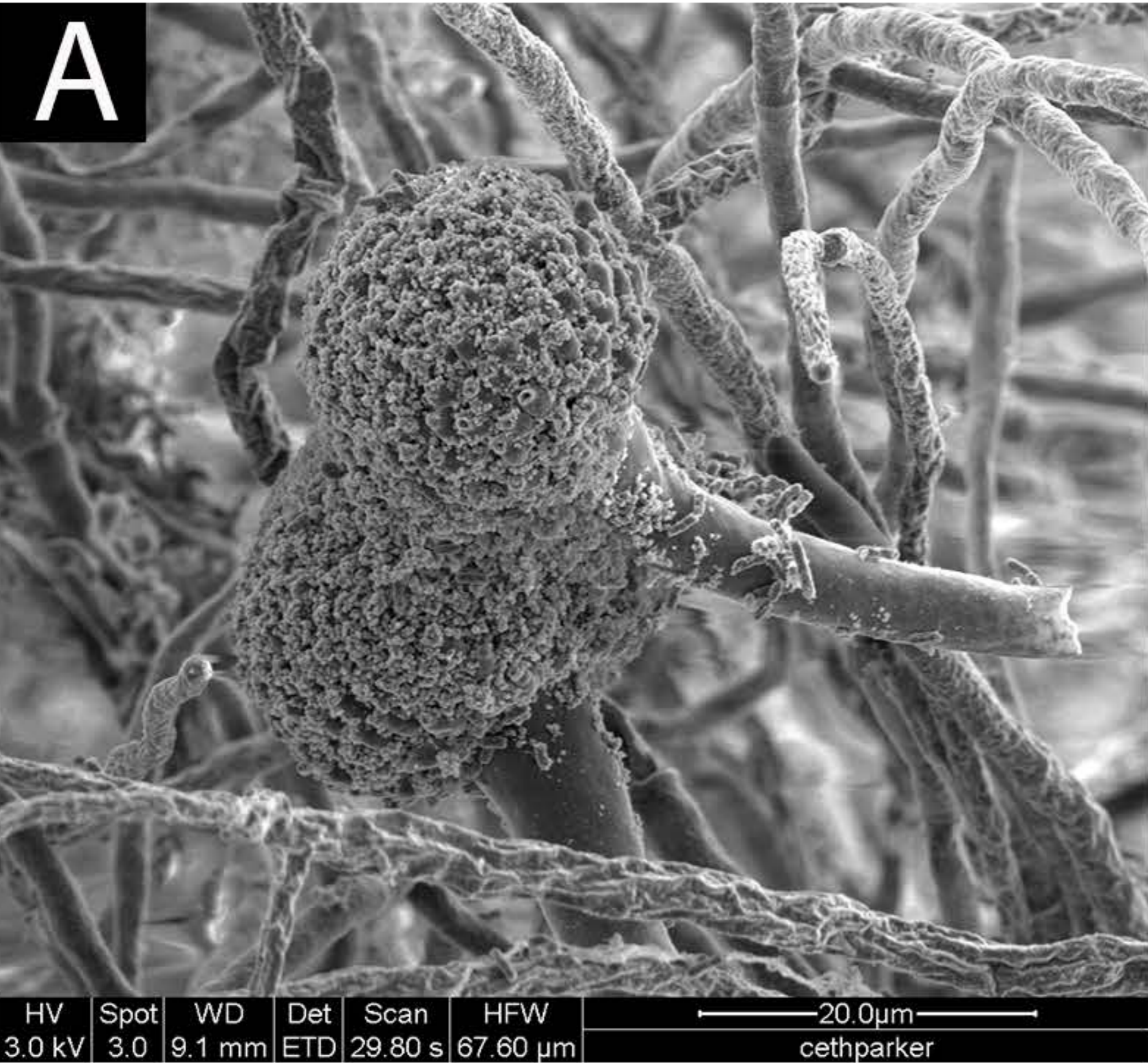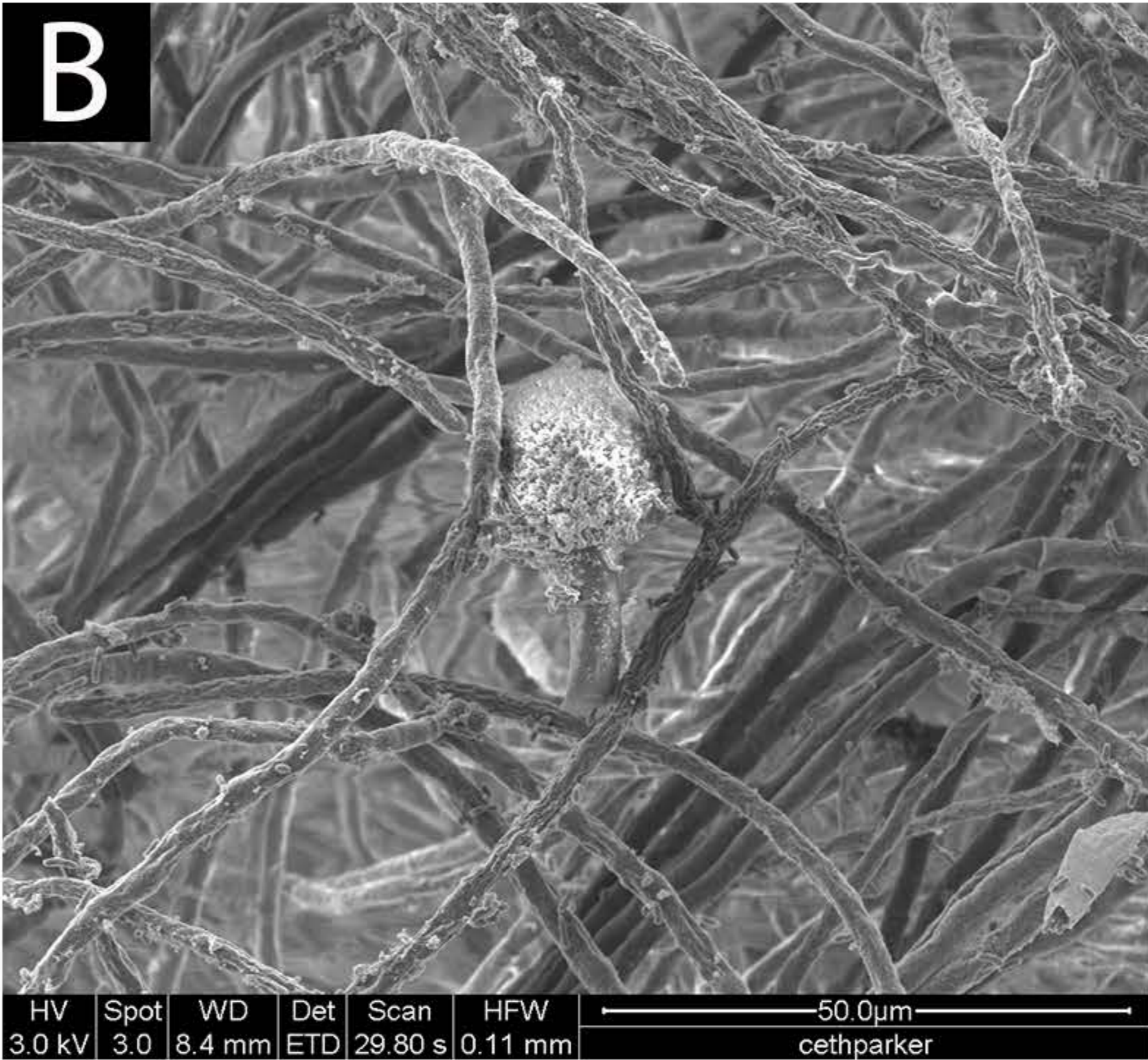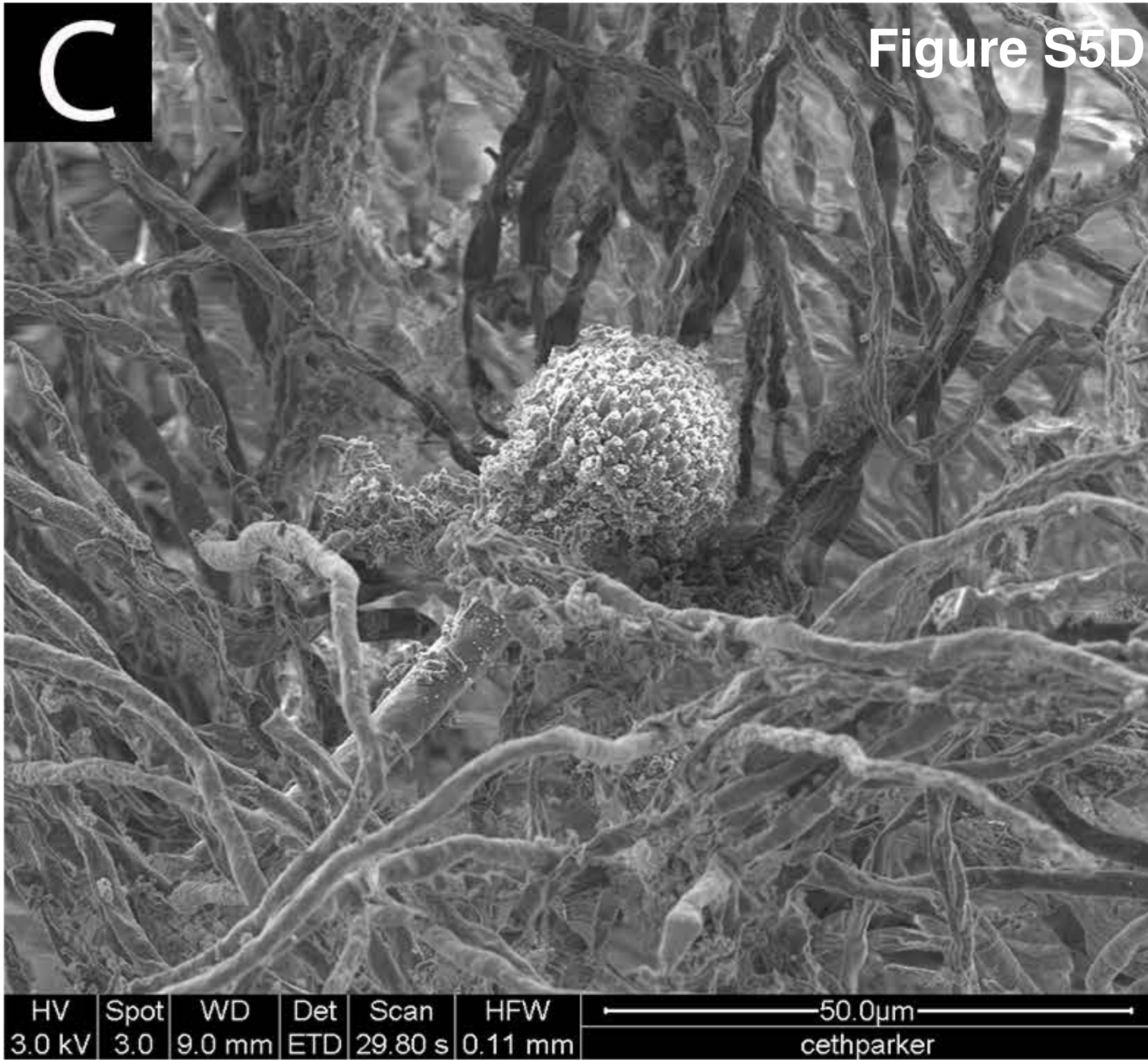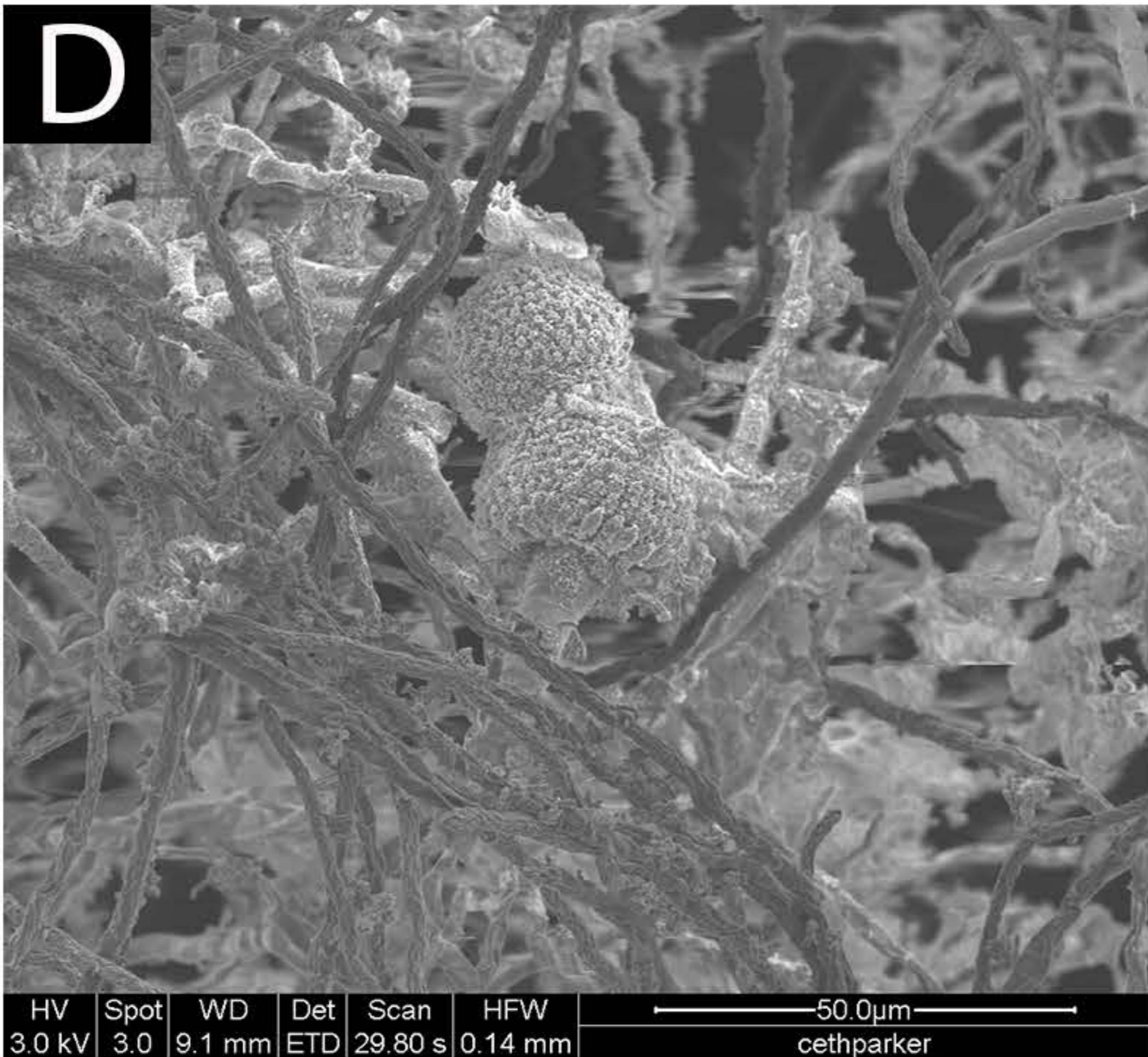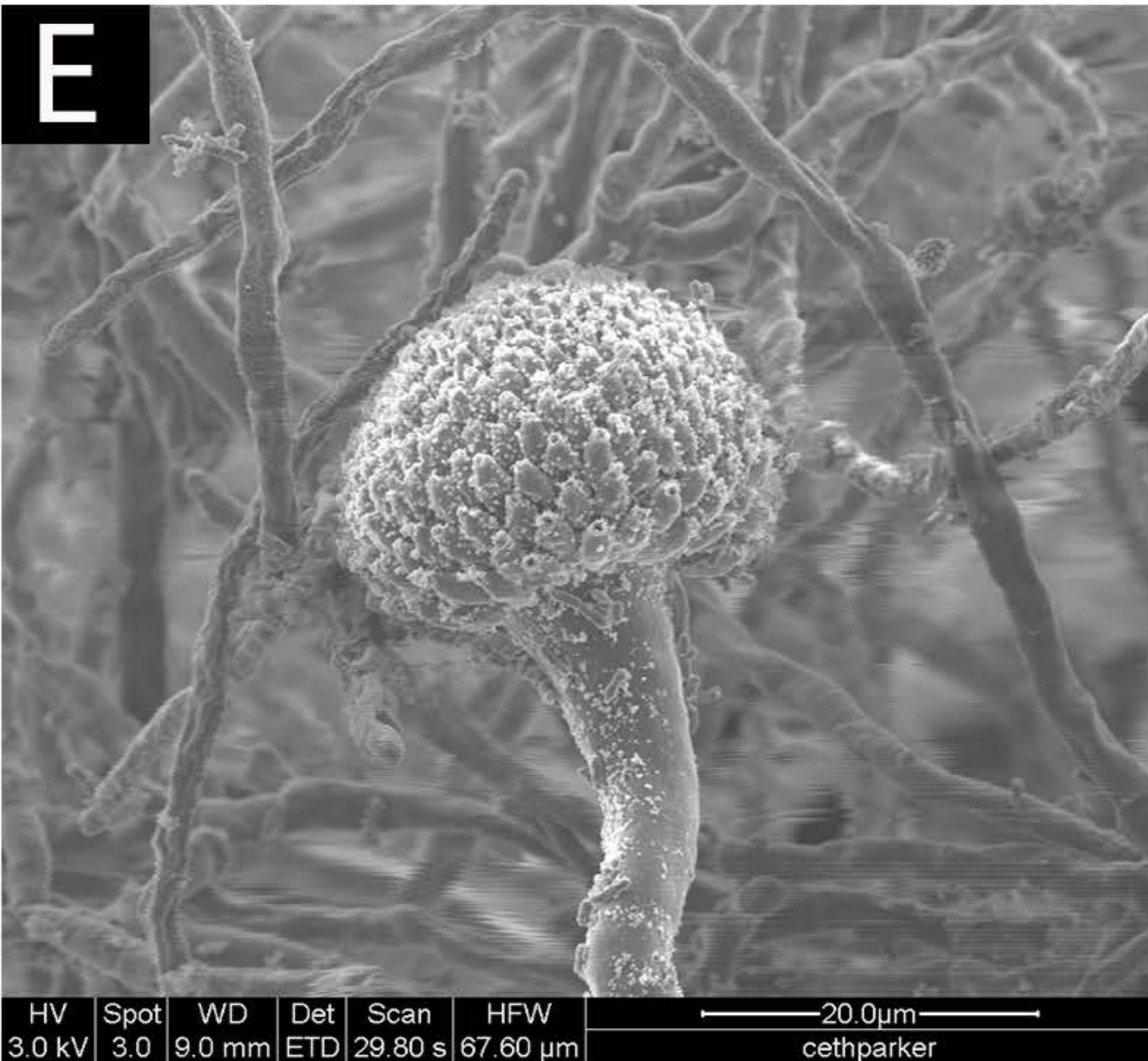

Supplement: Supplementary file 7 — Additional file 6: Supplementary Figure S5. Morphological architecture of A. fumigatus in the presence and absence of K. pneumoniae. (A) A. fumigatus grown under normal gravity, (B) Both bacteria and fungus grown under normal gravity, (C) A. fumigatus grown under simulated gravity, and (D) Both bacteria and fungus grown under simulated gravity. These pictures are representative micrographs of hundreds of SEM images. [file 40168_2022_1279_MOESM6_ESM.pdf]

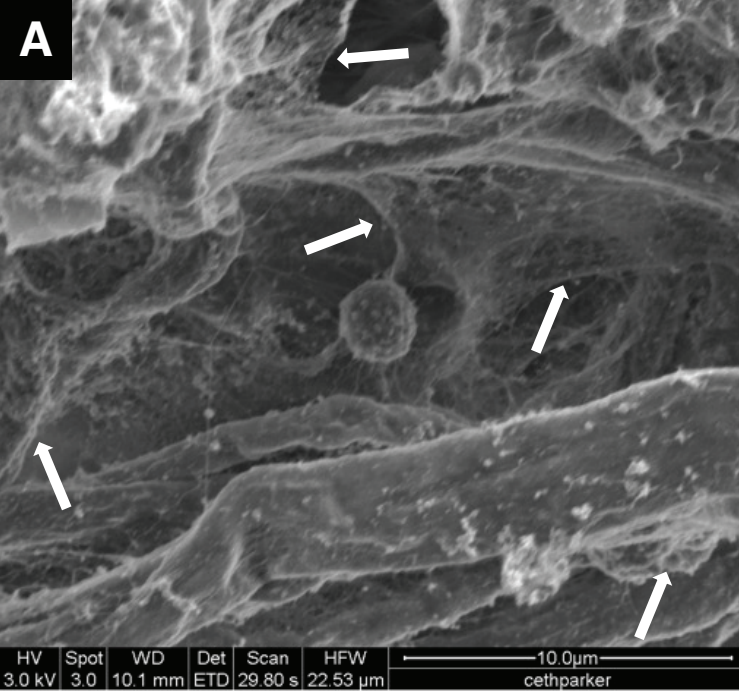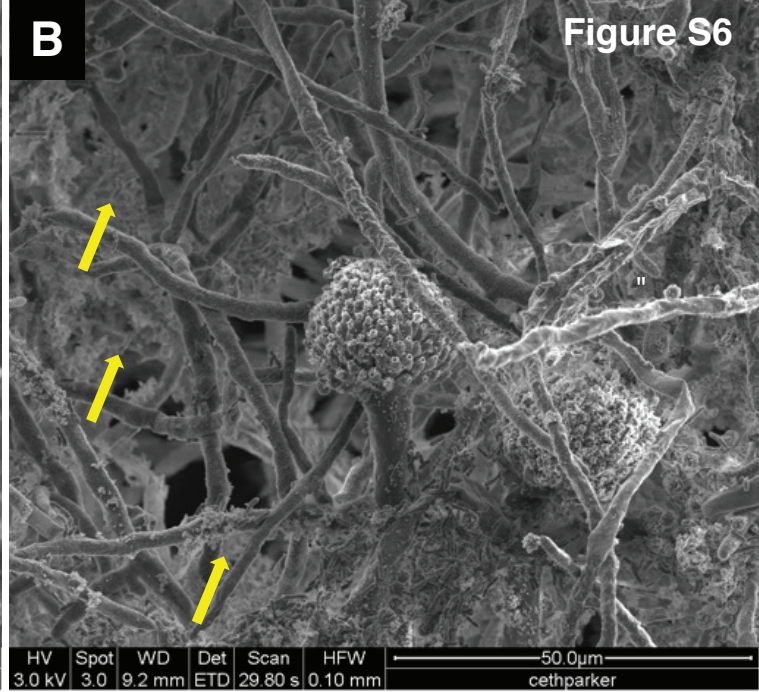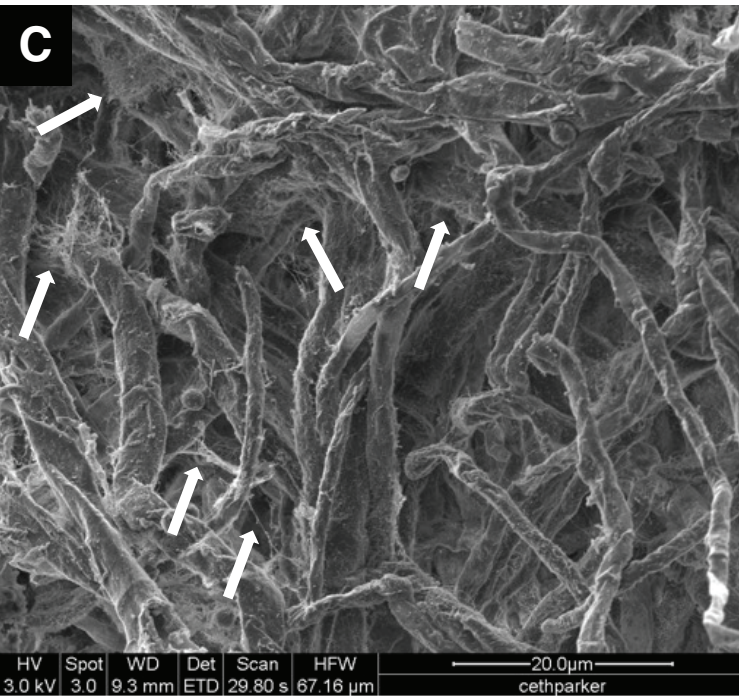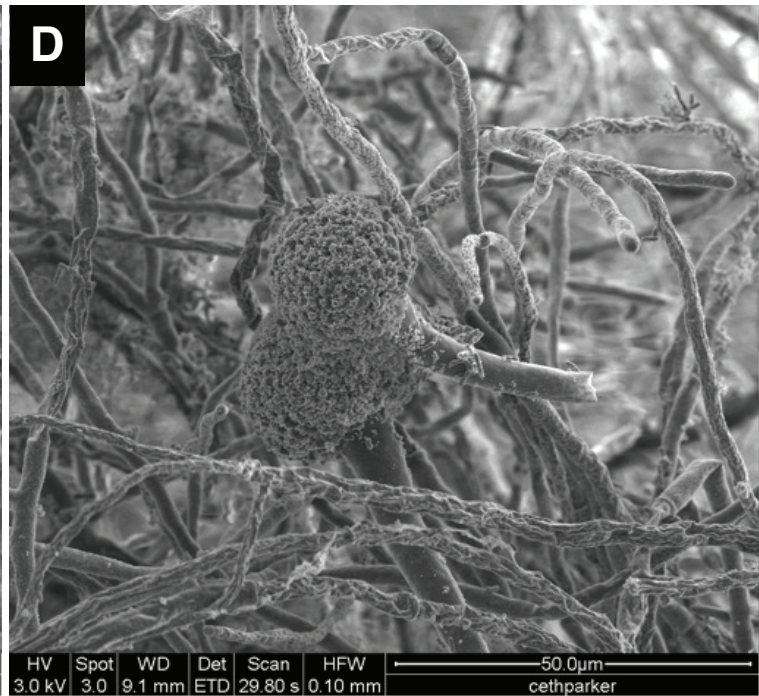

Supplement: Supplementary file 8 — Additional file 7: Supplementary Figure S6. Polymeric filamentous structures of A. fumigatus when co-cultured with K. pneumoniae under simulated microgravity. (A) A. fumigatus grown under normal gravity, (B) Both bacteria and fungus grown under normal gravity, (C) A. fumigatus grown under simulated gravity, and (D) Both bacteria and fungus grown under simulated gravity. White arrows shown in A and C were speculated to be the polymeric filamentous structures that might enable the fungus to form biofilm. However, when K. pneumoniae cells co-cultured with A. fumigatus (B and D) polymeric filamentous structures were not noticed. Yellow arrows were speculated to be the degraded fungal products by K. pneumoniae cells. When both bacteria and fungus grown under normal (B) or simulated gravity (D), the polymeric substances are not observed. However as shown in panel D, these structures are virtually not present. These pictures are the representative micrographs of hundreds of SEM images. [file 40168_2022_1279_MOESM7_ESM.pdf]
